# Immunogenicity of concomitant SARS-CoV-2 and influenza vaccination in UK healthcare workers: a prospective longitudinal observational study

**DOI:** 10.1016/j.lanepe.2024.101022

**Published:** 2024-08-12

**Authors:** Joshua Nazareth, Christopher A. Martin, Daniel Pan, Ian G. Barr, Sheena G. Sullivan, Heidi Peck, Neyme Veli, Mrinal Das, Luke Bryant, Nisha George, Marjan Gohar, Laura J. Gray, Lucy Teece, Denny Vail, Val Renals, Aleesha Karia, Paul Renals, Paul Moss, Andrea Tattersall, Ashley D. Otter, Pranab Haldar, Andrea Cooper, Iain Stephenson, Martin J. Wiselka, Julian W. Tang, Laura Nellums, Manish Pareek

**Affiliations:** a Department of Respiratory Sciences, University of Leicester, UK; b Department of Infection and HIV Medicine, University Hospitals of Leicester NHS Trust, Leicester, UK; c Leicester NIHR Biomedical Research Centre, Leicester, UK; d Development Centre for Population Health, University of Leicester, Leicester, UK; e Li Ka Shing Centre for Health Information and Discovery, Oxford Big Data Institute, University of Oxford, UK; f WHO Collaborating Centre for Infectious Disease Epidemiology and Control, School of Public Health, Li Ka Shing Faculty of Medicine, The University of Hong Kong, Hong Kong, China; g WHO Collaborating Centre for Reference and Research on Influenza, Royal Melbourne Hospital, at The Peter Doherty Institute for Infection and Immunity, Melbourne, Australia; h Department of Infectious Diseases, University of Melbourne, Melbourne, Australia; i Department of Epidemiology, University of California, Los Angeles, USA; j Department of Population Health Sciences, University of Leicester, UK; k Research Space, University Hospitals of Leicester NHS Trust, UK; l Institute of Immunology and Immunotherapy, University of Birmingham, Birmingham, UK; m Revvity (Certimmune), Abingdon, Oxfordshire, UK; n UK Health Security Agency, Porton Down, Salisbury, UK; o Department of Respiratory Medicine, University Hospitals of Leicester NHS Trust, Leicester, UK; p Department of Clinical Microbiology, University Hospitals of Leicester NHS Trust, Leicester, UK; q Lifespan and Population Health Academic Unit, School of Medicine, University of Nottingham, Nottingham, UK; r College of Population Health, University of New Mexico, NM, USA

**Keywords:** SARS-CoV-2, Influenza, Vaccine co-administration, Immunogenicity

## Abstract

**Background:**

Co-administration of inactivated influenza vaccine (IIV) and SARS-CoV-2 vaccine may impact SARS-CoV-2 vaccine induced humoral immune responses. We aimed to compare IIV and SARS-CoV-2 vaccine induced cellular and humoral immune responses in those receiving concomitant vaccination to those receiving these vaccines separately.

**Methods:**

We conducted a cohort study between 29th September 2021 and 5th August 2022 in healthcare workers who worked at the local NHS trust and in the surrounding area that were vaccinated with a mRNA SARS-CoV-2 booster and cell-based IIV. We measured haemagglutination inhibition assay (HAI) titres, SARS-CoV-2 anti-spike antibody and SARS-CoV-2 ELISpot count pre-vaccination, 1-month and 6-months post-vaccination and evaluated differences by vaccine strategy.

**Findings:**

We recruited 420 participants, 234/420 (56%) were vaccinated concomitantly and 186/420 (44%) separately. The 1-month post-vaccination mean fold rise (MFR) in SARS-CoV-2 anti-spike antibodies was lower in those vaccinated concomitantly compared to separately (MFR [95% confidence interval (CI)] 9.7 [8.3, 11.4] vs 12.8 [10.3, 15.9], *p* = 0.04). After adjustment for age and sex, the adjusted geometric mean ratio (aGMR) remained lower for those vaccinated concomitantly compared to separately (aGMR [95% CI] 0.80 [0.70, 0.92], *p* = 0.001). At 6-months post-vaccination, we found no statistically significant difference in SARS-CoV-2 anti-spike antibody titres (aGMR [95% CI] 1.09 [0.87, 1.35], *p* = 0.45). We found no statistically significant correlation between vaccine strategy with SARS-CoV-2 ELISpot count and influenza HAI titres at 1-month and 6-months post-vaccination.

**Interpretation:**

Our study found that concomitant vaccination with SARS-CoV-2 and IIV has no statistically significant impacts on long-term immunogenicity. Further research is required to understand the underlying mechanisms and assess the clinical significance of reduced anti-spike antibodies in those vaccinated concomitantly.

**Funding:**

Research and Innovation (UKRI) through the COVID-19 National Core Studies Immunity (NCSi) programme (MC_PC_20060).

**Research in context**

**Evidence before this study**

Vaccination against influenza and SARS-CoV-2 reduces the risk of developing severe disease from these infections. Co-administrating both vaccines may increase vaccine uptake and reduce logistical barriers to implementation. We searched PubMed for research articles published from database inception to August 1st 2023, with no language restrictions using the terms “SARS-CoV-2” or “COVID-19”, “influenza”, “vaccine”, “timing”, “co-administration” or “concomitant”, and “immunogenicity”. We identified five randomised controlled trials and two observational studies from the existing literature that evaluated immunogenicity following co-administration of a number of different SARS-CoV-2 vaccines (mRNA, subunit vaccines and inactivated) with cell-grown and egg-grown inactivated influenza vaccines. Some studies did not identify any differences between those vaccinated concomitantly and separately and one found lower influenza antibody titres in those vaccinated separately. Several other studies have found lower SARS-CoV-2 antibody titres in those vaccinated concomitantly. Of note, all these studies evaluated serology outcomes and not cellular immune responses. In addition, all studies only assessed antibody titres in the four to six weeks following vaccination.

**Added value of this study**

Our study adds to the growing body of evidence that anti-spike antibody titres to SARS-CoV-2 are lower in individuals vaccinated concomitantly with the inactivated influenza vaccine post-vaccination. In contrast, T cell responses to SARS-CoV-2 and influenza antibody titres were unaffected by vaccine strategy. To our knowledge this is the first study to show that haemagglutination inhibition assay antibody titres, SARS-CoV-2 cellular and humoral immune response are unaffected by concomitant vaccination at 6-months post-vaccination.

**Implications of all the available evidence**

Concomitant vaccination maximises vaccine uptake, without any detrimental effects on cellular immune responses to SARS-CoV-2 and serological responses to influenza. The finding of a reduced SARS-CoV-2 humoral immune response 1-month after both vaccines are given concomitantly requires further research to assess the impact this has on vaccine effectiveness. The effect of vaccination at separate times in relation to vaccine uptake should also be assessed to inform potential changes to vaccine schedules.

## Introduction

Influenza and SARS-CoV-2 persist as significant sources of global morbidity and mortality.^1^^,^^2^ Vaccines have been developed for both infections that prevent severe disease. Annual vaccination for influenza and SARS-CoV-2 is recommended for at-risk groups in many countries. This is to ensure circulating viruses closely match those contained in the vaccine, and due to waning of vaccine-induced immunity.^3^^,^^4^

In temperate climates, the incidence of respiratory virus infections peak during the winter months.^5^ Vaccination for both influenza and SARS-CoV-2 are therefore offered in the autumn, prior to periods of high respiratory virus circulation. When SARS-CoV-2 vaccines initially became available, national guidelines recommended against concomitant vaccination with the inactivated influenza vaccine (IIV) to avoid difficulty in attributing side-effects to a specific vaccine.^6^ Following the initial results of randomised control trials investigating the impact of co-administration on reactogenicity and immunogenicity that showed no significant differences when the two vaccines were given together,^7^^,^^8^ concomitant vaccination is now recommended and offered in many countries to reduce the number of vaccine-related visits, reduce pressure on healthcare services and increase vaccine coverage.^9^^,^^10^ As further data have accumulated, it has become clear that co-administrating the influenza vaccine with the SARS-CoV-2 vaccine can have an impact on immunogenicity in the immediate post-vaccination period.^11^, ^12^, ^13^, ^14^ However, there is limited evidence for the impact of concomitant vaccination on the longer-term durability of antibodies and the cellular immune response.

We aimed to compare, in UK healthcare workers (HCWs), SARS-CoV-2 anti-spike antibody titres, SARS-CoV-2 ELISpot count and influenza haemagglutination inhibition (HAI) assay titres at 1-month post-vaccination in those vaccinated with the IIV and SARS-CoV-2 vaccine concomitantly and in those receiving the vaccines separately. Our secondary aim was to compare these same immune parameters at 6-months post-vaccination in those vaccinated concomitantly and separately.

## Methods

### Study design

This study was conducted at University Hospitals of Leicester NHS Trust (UHL) as part of an ongoing single-centre prospective cohort study (see Supplementary Text 1 for further details). We conducted this component of the prospective cohort study between 29th September 2021 and 5th August 2022. We recruited HCWs aged 16 years or over who worked at UHL and in the surrounding area. HCWs could participate regardless of previous influenza and SARS-CoV-2 infections or vaccinations. For the purpose of this study, we used the term HCW to include any staff member with or without direct patient contact, as well as healthcare students and volunteers.

The study was advertised to HCWs that had taken part in previous observational studies at the Trust, including BELIEVE (Broadening our understanding of Early vs Late InfluEnza Vaccine Effectiveness) and DIRECT (Determining the Immune Response in Ethnic minority healthcare workers to COVID-19 infecTion). Additional details regarding recruitment to these studies have been published elsewhere.^4^^,^^15^ The BE-DIRECT study was also advertised in hospital-wide email communications and on the staff intranet. This was supplemented by direct recruitment from clinical and non-clinical areas of the hospital. Sample size calculations were not performed for this observational study.

### Study visits

After providing written informed consent, participants provided information on demographic and occupational characteristics. Blood samples were taken at the following timepoints: 1) Pre-SARS-CoV-2 booster and IIV; 2) 1-month post-SARS-CoV-2 booster; 3) 1-month post-IIV; and 4) Approximately 6-months post-IIV and SARS-CoV-2 booster (See Supplementary Figure S1 for further details). We also asked participants to notify the study team if they had a positive SARS-CoV-2 or influenza test during the study period, but we did not conduct active surveillance of the cohort.

### Demographic and clinical data

We collected information on self-reported ethnicity as defined by the Office National Statistics (ONS),^16^ age in years, sex, dates of the two previous SARS-CoV-2 vaccine doses received and number of IIV doses received in the last four years.

### Vaccines

This was an observational study; participants were free to decide if they wanted to receive the IIV and SARS-CoV-2 vaccine separately or concomitantly. For this study concomitant vaccination was defined as vaccination on the same day; vaccination with a one day or more separation was regarded as separate vaccination. Participants were vaccinated with an mRNA SARS-CoV-2 vaccine (BNT162b2/Comirnaty, Pfizer-BioNTech). This was the third dose of SARS-CoV-2 vaccine participants received and in this study we have used the term SARS-CoV-2 booster to describe this vaccine dose. Participants also received cell-based quadrivalent IIV (Flucelvax Tetra; manufactured using a proprietary Madin Darby Canine Kidney (MDCK) cell line, inactivated split virion, containing 15 μg haemagglutinin for each influenza antigen per 0.5 ml dose, Seqirus Vaccines Limited). The 2021/2022 IIV included four influenza antigens following WHO recommendations: A/Wisconsin/588/2019 (H1N1)pdm09-like strain, A/Cambodia/e0826360/2020 (H3N2)-like strain, B/Washington/02/2019-like strain and B/Phuket/3073/2013-like strain.^17^ The same vaccines were used in the rest of the healthcare trust as part of the broader national vaccination programme.

### Laboratory methods

#### SARS-CoV-2 serology assay

Anti-spike and anti-nucleocapsid SARS-CoV-2 serology were performed at UKHSA Porton Down on serum samples using the Roche Elecsys anti-SARS-CoV-2 S (Product code: 09203079190) and Roche Elecsys anti-SARS-CoV-2 (Product code: 09289275190) assays (Supplementary Text 2), which utilise the Wuhan receptor binding domain and so were homologous to the mRNA SARS-CoV-2 vaccine (BNT162b2/Comirnaty, Pfizer-BioNTech). Samples were considered positive for anti-spike antibodies if ≥0.8 binding antibody units per millilitre (BAU/ml), and positive for anti-nucleocapsid antibodies if ≥1 cutoff index (COI).

#### SARS-CoV-2 ELISpot assay

To quantify SARS-CoV-2 T cell responses, we used T-SPOT® Discovery SARS-CoV-2 platform (Revvity), which use ELISpot technology to detect IFN-γ release from immune cells after stimulation with SARS-CoV-2 peptides. Peripheral blood mononuclear cells were isolated within 32 h of test performance. The T-SPOT Discovery SARS-CoV-2 test was performed according to the instructions of the kit and as described previously,^15^ and we present responses to the spike peptides Spike 1 (S1), Spike 2 (S2) and Spike (S1 + S2).

#### Influenza haemagglutination inhibition (HAI) assay

The HAI assays were performed at the World Health Organization (WHO) Collaborating Centre for Reference and Research on Influenza, Melbourne, Australia according to the WHO method (see Supplementary Text 3 for further details). Viruses used for the HAI assay were identical to those used in the vaccine. The serum titre was expressed as the reciprocal of the highest serum dilution that caused complete inhibition of hemagglutination.

### Statistical analysis

We aimed to examine the immune responses to the SARS-CoV-2 booster and IIV. We first excluded individuals from the analysis who did not receive both these vaccinations during the study period and those that had not previously received a primary course of two doses of a SARS-CoV-2 vaccine. Secondly, since our study focuses on the immune responses to vaccination and not infection, we excluded participants that had evidence of SARS-CoV-2 and/or influenza infection from the respective analysis of SARS-CoV-2 and influenza immune responses. As with previous influenza longitudinal studies, we considered evidence of influenza infection as a 4-fold or greater rise in HAI titre between 1-month and 6-months post-vaccination sera from the same individual,^18^ as well as self-reported PCR positive test. We excluded HCWs who reported a positive SARS-CoV-2 test during the study period. Additionally, we defined evidence of SARS-CoV-2 infection by changes in anti-nucleocapsid and ELISpot count (see Supplementary Text 4 for further details).

We summarised categorical variables as frequency and percent, normally distributed continuous variables as mean and standard deviation (s.d.) and non-normally distributed continuous variables as median and interquartile range (IQR). Continuous variables were assessed for normality of distribution by visual inspection. To compare differences in demographic and clinical features between the two vaccine strategy groups we used Student's *t* test for parametric continuous variables, Wilcoxon rank-sum test for non-parametric continuous variables and chi-squared tests for categorical variables. The variables of interest in our study had minimal missing data, eliminating the need for imputation or additional strategies to address this issue.

HAI and anti-spike antibody titres were log_2_ transformed prior to analysis. Raw ELISpot counts were transformed first by subtracting the count from a nil control and then multiplied by four to give a value in spot forming units (SFUs) per million peripheral blood mononuclear cells (PBMCs) (See Supplementary text 5 for more details).^15^

To assess the initial boost in antibody titres following vaccination we calculated the HAI and anti-spike antibody geometric mean fold rises (MFRs) with 95% confidence intervals (CIs). Similarly, to assess the decline in antibody titres after vaccination we calculated HAI and anti-spike antibody MFRs between 1-month and 6-months post-vaccination. For unadjusted comparisons of immune parameters between those vaccinated separately and concomitantly, we used Student's *t* test to compare MFRs and Wilcoxon rank-sum test to compare ELISpot counts.

We used multivariable linear regression to determine the effects of concomitant vaccination on HAI and anti-spike antibody levels after adjustment for pre-vaccination antibody titre and differences in baseline characteristics that were statistically significant between those vaccinated separately or at the same time. We also used multivariable linear regression to determine the effects of concomitant vaccination on HAI and anti-spike antibody levels at 6-months post-vaccination after adjustment for differences in baseline characteristics and antibody titre 1-month post-vaccination. To calculate adjusted geometric mean ratio (aGMR), the natural logarithm of antibody titres was used in the multivariable models. Coefficients of vaccine titres used as independent variables were therefore presented as per *e* or ∼2.72-fold increase.

In these multivariable linear regression models, where vaccination strategy was a significant predictor of either influenza or SARS-CoV-2 antibody titre, we conducted further sub-group analyses. Firstly, we included only those participants that were vaccinated separately, and used the number of weeks between the influenza and SARS-CoV-2 vaccines as a continuous predictor variable to assess if the length of time between the two vaccinations has an impact on post-vaccine antibody titres. Secondly, we performed a sub-group analysis using separate linear regression models to compare those vaccinated concomitantly with participants vaccinated with the IIV before the SARS-CoV-2 booster and participants that received the first SARS-CoV-2 booster followed by the IIV to explore if the order of vaccinations impacted on antibody titre. Thirdly, we performed a sub-group analysis at 1-month post-booster restricted to participants that did not become infected throughout the duration of the six months follow-up.

As in our previous work, we used negative binomial regression to investigate the impact of concomitant vaccination on ELISpot count 1-month and 6-months post-vaccination after adjustment for the same variables used in the linear regression models.^15^ Results were expressed as adjusted incidence rate ratios (aIRRs).

All analyses were performed using Stata 17 (StataCorp. 2021. Stata Statistical Software: Release 17. College Station, TX: StataCorp LLC.)^19^ Figures were created in GraphPad Prism version 9.4.1 for macOS (GraphPad Software, San Diego, California USA, www.graphpad.com). We considered *p* values < 0.05 to be statistically significant.

### Ethical approval

BE-DIRECT was approved by the Health Research Authority (Brighton and Sussex Research Ethics Committee; ethics reference: 20/HRA/4718). All participants gave informed consent.

### Role of the funding source

The study was funded by Research and Innovation (UKRI) through the COVID-19 National Core Studies Immunity (NCSi) programme (MC_PC_20060). The funders had no role in study design, data collection, data analysis, interpretation, writing of the report or decision to submit the paper for publication.

## Results

### Description of the cohort

**Fig. 1:**
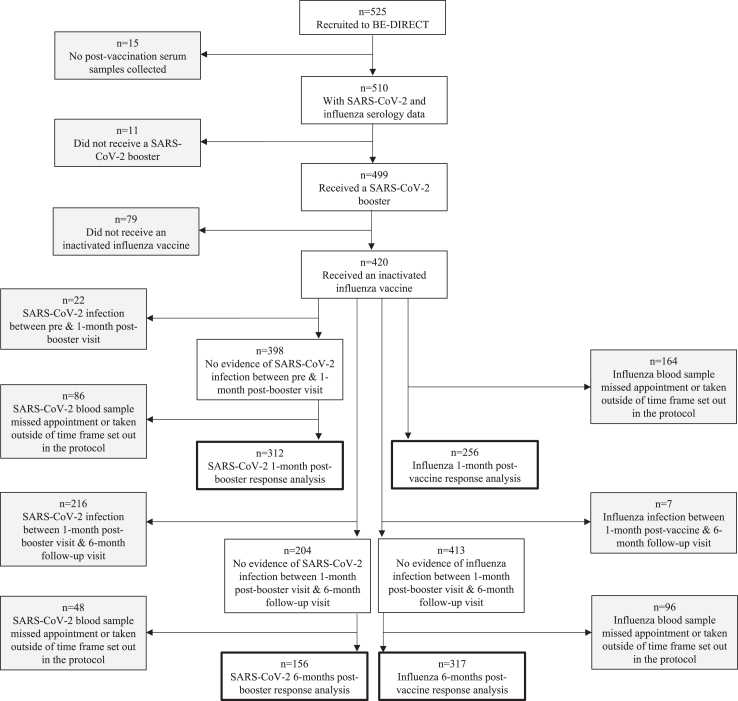
**Flowchart illustrating the analysed cohort**. Flowchart of study participants included in the analysed cohort: 1-month post-vaccine and 6-months postvaccine for influenza immune responses and 1-month post-booster and 6-months post-booster for SARS-CoV-2 immune responses.

Fig. 1 shows the formation of the analysed cohort and the number of participants included in each of the analyses. Of the 420 participants included in the analysis 234 (56%) were vaccinated concomitantly and 186 (44%) received the influenza and SARS-CoV-2 vaccination separately. There was 62 (33%) of participants that received the IIV before the SARS-CoV-2 booster, and 124 (67%) received the IIV after the SARS-CoV-2 booster. Participants received the vaccines between 29th September 2021 and 14th February 2022. Table 1 summarises the baseline characteristics of participants for each vaccination strategy. Demographics reflected that of UHL and the wider NHS workforce.^20^ Supplementary Table S1 shows baseline characteristics for each of the four analyses: 1-month post-vaccine and 6-months post-vaccine for influenza immune responses and 1-month post-booster and 6-months post-booster for SARS-CoV-2 immune responses. Supplementary Table S2 shows baseline characteristics of the participants included and excluded in each analysis.

**Table 1:** Baseline characteristics of the cohort.

	Vaccinated concomitantly n = 234	Vaccinated separately n = 186	*p* value	Total n = 420	Missing data (%)
**Demographic details**					
Age in Years					
Median (IQR)	42 (32, 51)	47 (35, 56)	0.002	44 (33, 53)	0
Sex					
Female	190 (81)	167 (90)	0.01	357 (85)	0
Male	44 (19)	19 (10)		63 (15)	
Ethnicity					
White	188 (80)	140 (75)	0.44	328 (78)	0
South Asian	33 (14)	35 (19)		68 (16)	
Black/Mixed/Other	13 (6)	11 (6)		24 (6)	
**Clinical features**					
Patient Facing	117 (50)	107 (58)	0.13	224 (53)	0
Non-Patient Facing	117 (50)	79 (42)		196 (47)	
Weeks between SAR-CoV-2 booster and influenza vaccination (IQR)	0	3.2 (1.7, 5.3)	NA	NA	0
Weeks between influenza vaccine and 1-month post-vaccine sample (IQR)	3.2 (3, 4)	3.3 (3, 4)	0.46	3.3 (3, 4)	0
Weeks between influenza vaccine and 6-months post-vaccine sample (IQR)	31 (29, 34)	31 (29, 35)	0.93	31 (29, 35)	0
Weeks between SARS-CoV-2 booster and 1-month post-booster sample (IQR)	3.4 (3, 4)	3.6 (3, 4)	0.76	3.4 (3, 4)	0
Weeks between SARS-CoV-2 booster and 6-months post-booster sample (IQR)	31 (29, 37)	34 (29, 38)	0.90	32 (29, 37)	0
SARS-CoV-2 infection pre- to 1-month post-booster	14 (6)	8 (4)	0.43	22 (5)	0
SARS-CoV-2 infection 1-month post-booster to 6-month post-booster follow-up	123 (53)	93 (50)	0.12	216 (58)	0
Influenza infection pre-booster to 6-month post-IIV	2 (1)	5 (3)	0.15	7 (2)	0
Number of influenza vaccines received in the previous 4 years					
0	11 (6)	6 (4)	0.48	17 (5)	99 (24)
1	16 (9)	8 (5)		24 (7)	
2	9 (5)	9 (6)		18 (6)	
3	11 (6)	14 (9)		25 (8)	
4	126 (73)	111 (75)		237 (74)	

Baseline characteristics of the participants included in the analysis (n = 420) displayed for the overall group and for each vaccination strategy, influenza vaccine and SARS-CoV-2 booster administered concomitantly or separately. All results are shown as n (%) unless otherwise stated with percentage being presented column-wise. Differences between the two vaccine strategy groups were compared using Wilcoxon rank-sum tests and chi-squared tests for continuous and categorical variables respectively. Participants with missing samples in any of the analyses of interest were excluded, see Supplementary Table S1 for details of participants included in each of the four analyses: 1-month post-vaccine and 6-months post-vaccine for influenza immune responses and 1-month post-booster and 6-months post-booster for SARS-CoV-2 immune responses. For the number of influenza vaccines received in the previous 4 years, participants were asked about their influenza vaccination status in each of the last four years, any participant who responded ‘unsure’ in any of the previous four years was then defined as missing. IQR, Interquartile range; IIV, inactivated influenza vaccine; NA, Not applicable.

Compared to those vaccinated separately, participants that received the vaccine concomitantly were younger (median (IQR) 42 years (32–51), vs 47 years (35–56) *p* = 0.002) and a greater proportion were males (44/234 19% vs 19/186 10%, *p* = 0.01). For those vaccinated separately, the median duration between SARS-CoV-2 and influenza vaccination was 22 days (IQR 11–37). Participants vaccinated with the IIV first received the SARS-CoV-2 booster a median of 21 days (IQR 11–38) after. Participants vaccinated with the SARS-CoV-2 booster first received the IIV a median of 24 days (IQR 12–37) after. We found no difference in the number of breakthrough SARS-CoV-2 and influenza infections during the study period by vaccine strategy (SARS-CoV-2 infection 1-month post-booster to 6-month post-booster follow-up, concomitant 123/234 53% vs separate 93/186 50%, *p* = 0.12) (Table 1).

### SARS-CoV-2 serology

**Fig. 2:**
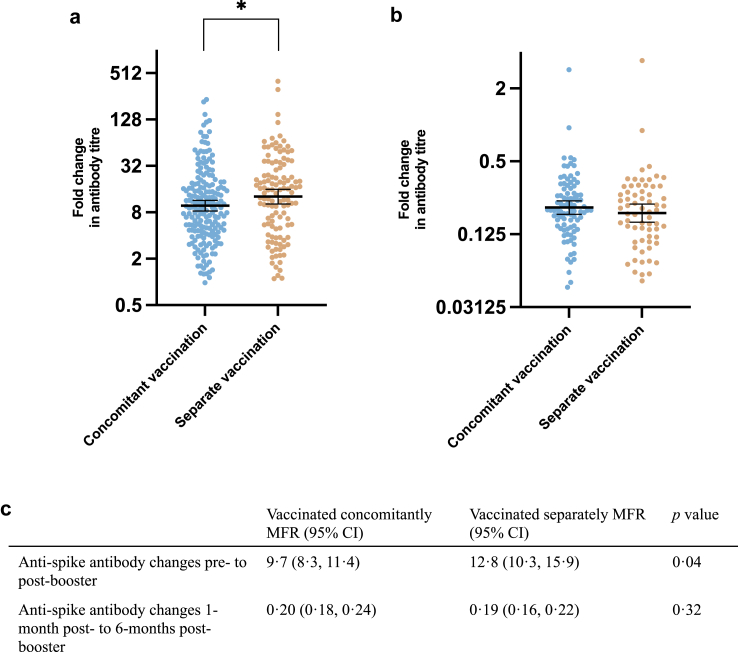
**Comparison of mean fold rise in SARS-CoV-2 anti-spike antibody titres by vaccine strategy**. Comparison of mean fold rise in SARS-CoV-2 anti-spike antibody titres between (a) pre- and 1-month post-booster and (b) 1-month post-booster to 6-months post-booster with the SARS-CoV-2 booster stratified by vaccine strategy. The table (c) shows the geometric mean fold rise and corresponding 95% confidence intervals between pre- and 1-month post-booster and 1-month post-booster to 6-months post-booster SARS-CoV-2 antispike antibody titres stratified by vaccine strategy. The two vaccine strategy groups were compared using an unpaired t test. MFR, mean fold rise; CI, confidence interval; ∗*p* value < 0.05.

The dot plot in Fig. 2 shows that SARS-CoV-2 anti-spike antibody MFR between pre- and post-booster was lower in those that received the vaccine concomitantly compared to those that were vaccinated separately (pre-to-1-month post-booster MFR [95% CI] 9.7 [8.3, 11.4] vs 12.8 [10.3, 15.9] *p* = 0.04). There was no difference in the MFR between the 1-month post and 6-months post vaccination visits by vaccine strategy (MFR [95% CI] 0.20 [0.18, 0.24] vs 0.19 [0.16, 0.22] *p* = 0.32).

**Fig. 3:**
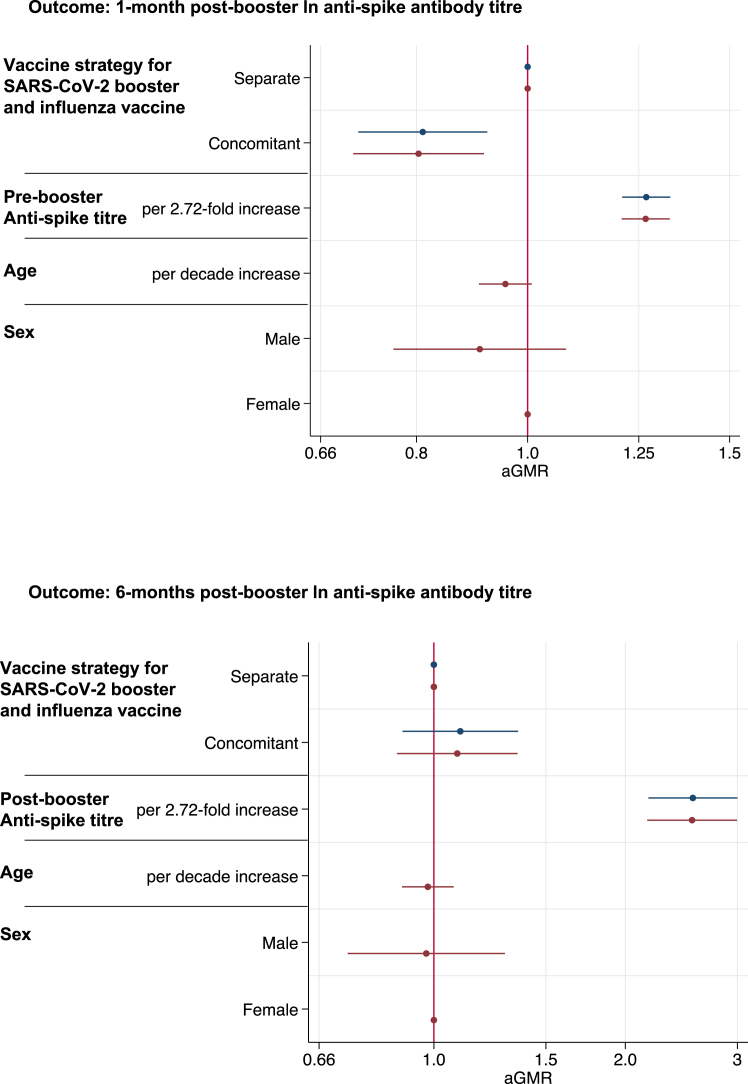
**Linear regression models showing the association between vaccine strategy and other parameters with ln SARS-CoV-2 post-booster anti-spike antibody titre**. Linear regression models showing the association between vaccine strategy, previous anti-spike antibody titre, age and sex with natural logarithm (ln) SARS-CoV-2 anti-spike 1-month and 6-months post-booster titres. Results are shown for a fully adjusted model (red) which included all variables and a simplified model (blue) which just adjusted for vaccine strategy and previous anti-spike titre.

Fig. 3 and Supplementary Table S3 show results from the multivariable linear regression model at 1-month and 6-months post-booster for anti-spike antibody titres. Anti-spike titres at the 1-month post-booster visit were reduced in participants that received the vaccines concomitantly, compared to those that received the vaccine separately, after adjusting for age and sex and pre-booster titre (aGMR [95% CI] 0.80 [0.70, 0.92] *p* = 0.001, with the participants vaccinated separately serving as the reference group). In those vaccinated separately, there was no association between the number of weeks between IIV and SARS-CoV-2 booster administration and post-booster SARS-CoV-2 anti-spike titre (aGMR per week increase [95% CI] 1.00 [0.96, 1.04] *p* = 0.93) (Supplementary Table S4). There was no significant difference in 6-months post-booster anti-spike titre for those vaccinated concomitantly (aGMR [95% CI] 1.09 [0.87, 1.35] *p* = 0.45) (Fig. 3 and Supplementary Table S3).

In the sub-group analysis SARS-CoV-2 anti-spike antibody titres were lower in those vaccinated concomitantly compared to participants that received the IIV before the SARS-CoV-2 booster (aGMR [95% CI] 0.74 [0.61, 0.90] *p* = 0.002) and were also lower compared to participants that received the IIV after the SARS-CoV-2 booster (aGMR [95% CI] 0.83 [0.72, 0.97] *p* = 0.02). In the sub-group analysis excluding participants that become infected with SARS-CoV-2 during the six months follow-up period, SARS-CoV-2 anti-spike antibody titres at 1-month post-booster were lower in those vaccinated concomitantly compared to separately (aGMR [95% CI] 0.80 [0.66, 0.96] *p* = 0.02) (Supplementary Table S5).

### SARS-CoV-2 ELISpot

Table 2 shows the spot counts using ELISpot following stimulation to S1 domain, S2 domain and total spike (S1 + S2) by vaccine strategy at the 1-month post-booster visit and at the 6-months post-booster visit. Spot counts to S1, S2 and spike did not differ by vaccination strategy at 1-month or 6-months post-booster.

**Table 2:** Comparison of T cell responses to SARS-CoV-2 S1, S2 and spike (S1 + S2) epitopes by vaccine strategy.

	Vaccinated concomitantly median (IQR)	Vaccinated separately median (IQR)	*p* value
**1-month post-booster**	n = 193	n = 119	
T cell S1	12 (5, 25)	15 (5, 31)	0.23
T cell S2	8 (4, 17)	10 (4, 20)	0.33
T cell Spike	23 (10, 40)	26 (11, 54)	0.25
**6-months post-booster**	n = 90	n = 66	
T cell S1	4 (2, 10)	5 (2, 12)	0.43
T cell S2	3 (1, 6)	3 (1, 10)	0.89
T cell Spike	9 (4, 19)	8 (3, 26)	0.63

Comparison of T cell responses using the ELISpot assay to SARS-CoV-2 S1, S2 and Spike (S1 + S2) epitopes by vaccine strategy. Median and interquartile range (IQR) spot forming units (SFU) per 10^6^ peripheral blood mononuclear cells (PBMCs) at 1-month post-booster and 6-months post-booster timepoints stratified by vaccine strategy. The two vaccine strategy groups were compared using a Wilcoxon rank sum test. IQR, interquartile range.

**Fig. 4:**
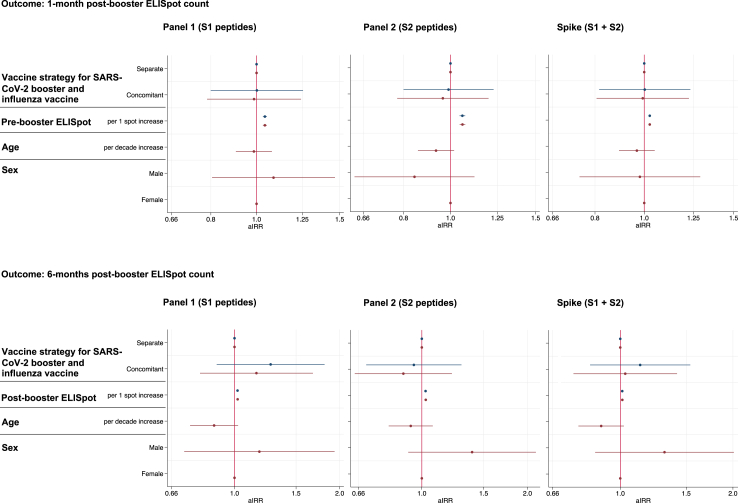
**Negative binomial regression model showing the association between vaccine strategy and other parameters with S1, S2 and Spike specific T cell responses**. Negative binomial regression models showing the association between vaccine strategy, previous ELISpot count, age and sex with ELISpot count in response to peptides derived from SARS-CoV-2 S1, S2 and spike (S1 + S2) 1-month post-booster and 6-months post-booster. Results are shown for a fully adjusted model (red) which included all variables and a simplified model (blue) which just adjusted for vaccine strategy and previous ELISpot count.

Fig. 4, Supplementary Tables S6 and S7 show the results of the multivariable negative binomial regression analysis of ELISpot counts in response to S1, S2 and spike peptides at both 1-month and 6-months respectively. There was no difference in ELISpot count by vaccine strategy after adjustment for age, sex and pre-booster or 1-month post-booster spot counts.

### Influenza serology

**Fig. 5:**
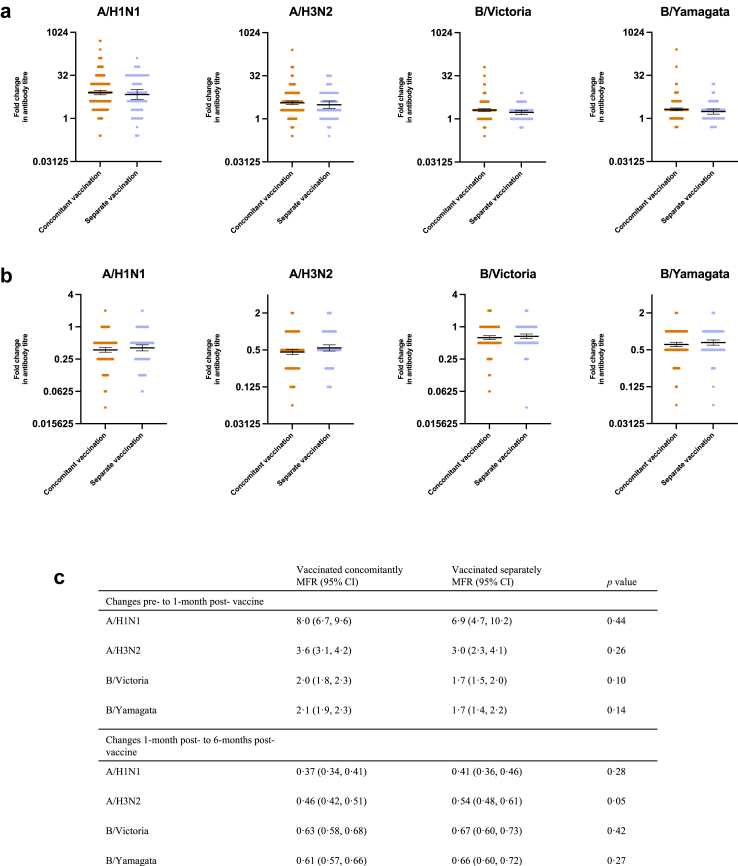
**Comparison of mean fold rise in HAI titres by vaccine strategy**. Comparison of mean fold rise in Haemagglutinin inhibition assay (HAI) titres between (a) pre- and 1- month post-vaccination and (b) 1-month and 6-months post-vaccination with the influenza vaccine stratified by vaccine strategy for each strain of influenza contained in the vaccine. Bars indicate the 95% CI, The table (c) shows the mean fold rise and corresponding 95% confidence intervals between pre- and post-vaccination and post-vaccination to 6-months post-vaccination HAI titres stratified by vaccine strategy for each strain of influenza. The two vaccine strategy groups were compared using an unpaired t test. MFR, mean fold rise; CI, confidence interval.

Dot plots showing the MFR by vaccination strategy between pre- and 1-month post-vaccination for each of the four influenza strains are shown in Fig. 5(a). MFR and statistical testing are summarised in Fig. 5. Fig. 5(b) also shows the dot plots of MFR and 95% CIs for changes between HAI titre at 6-months post-vaccination compared to the 1-month post-vaccination visit. There was no difference in mean fold rise for any of the influenza strains between those vaccinated concomitantly and separately.

**Fig. 6:**
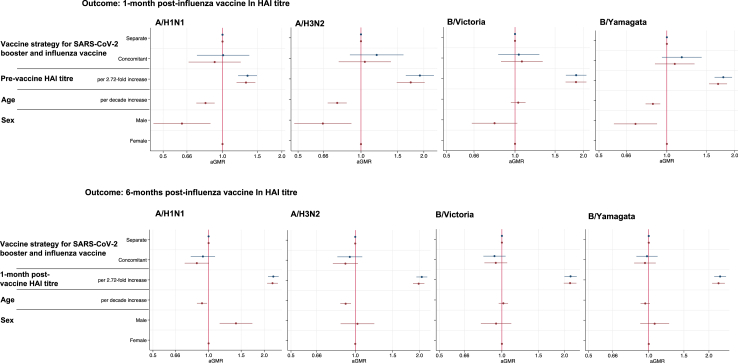
**Linear regression models showing the association between vaccine strategy and other parameters with ln HAI post-vaccine titre**. Linear regression models showing the association between vaccine strategy, previous haemagglutinin inhibition assay (HAI) titre, age and sex with natural logarithm (ln) HAI 1-month and 6-months post-vaccine titres for each influenza strain. Results are shown for a fully adjusted model (red) which included all variables and a simplified model (blue) which just adjusted for vaccine strategy and previous HAI titre.

Fig. 6, Supplementary Tables S8 and S9 show the results from the multivariable linear regression, again showing no difference in 1-month and 6-months post-vaccination HAI titre by vaccine strategy for all influenza strains.

## Discussion

We have shown that in a prospectively sampled, cohort of 420 HCWs there are differences in anti-spike antibody titres 1-month after a Pfizer BNT162b2 mRNA SARS-CoV-2 booster in those vaccinated concomitantly with the IIV compared to those vaccinated separately. After adjusting for age and sex we have shown that post-booster anti-spike titres are 20% lower in those vaccinated concomitantly compared to when the SARS-CoV-2 booster is administered at a separate time to the IIV. We have also shown that these differences in anti-spike antibody titres are not present 6-months after receiving the booster. Our analysis of ELISpot and influenza serology data found that there are no differences in SARS-CoV-2 cellular response and influenza HAI titres at 1-month and 6-months post-vaccinations.

These results are consistent with previous studies that have shown decreased anti-spike titres post-vaccination with the co-administration of IIV with the first dose of the SARS-CoV-2 primary vaccination series,^8^^,^^11^ and as a booster vaccination.^12^^,^^13^^,^^21^ Reduced antibody titres to vaccines given concomitantly with the IIV have also been reported for pneumococcal and meningococcal vaccines.^22^^,^^23^ Our results differ from initial randomised control trials evaluating IIV co-administration, which found co-administration with IIV had no impact on anti-spike titres when given with the SARS-CoV-2 vaccine in the primary course,^24^ and as a booster.^7^ It is unclear the reason for these differing findings. They may be due to between-study variation including the study population and the type of SARS-CoV-2 vaccination used. In this study all participants received the same Pfizer BNT162b2 mRNA booster so it is not clear if the same differences would be seen for other vaccine types. Other studies have seen a reduction in the SARS-CoV-2 serology peak response when IIV is administered with subunit vaccines (NVX-CoV2373),^8^ and inactivated vaccines (CoronaVac).^11^ Of note there are currently several influenza and SARS-CoV-2 vaccine coformulations in development which will also need to be evaluated to assess if immunogenicity is equivalent to separate vaccination.

Several potential mechanisms have been suggested to explain why concomitant vaccination may have an impact on immunogenicity, including: the induction of a type 1 interferon response by another vaccine leading to suppression of the immune response to mRNA vaccines; redirected vaccine imprinting due to another vaccine influencing the selection of B cell clones to other related sequences; and vaccine interference whereby there is an insufficient number of immune cells to elicit an adequate response to both vaccines.^25^, ^26^, ^27^

The clinical impact of a reduced peak anti-spike titre, of the magnitude seen in those vaccinated concomitantly in this study, is unclear. We did not report neutralising antibody titres against emerging variants, which may more closely correlate with protection against breakthrough infection,^13^^,^^28^ however, there is an established correlation between wild-type anti-spike antibody titres and neutralising titres to other SARS-CoV-2 variants.^29^^,^^30^ Dulfer and colleagues have also shown that SARS-CoV-2 and influenza vaccine co-administration has an impact on anti-spike antibodies as well as neutralising antibody titres against D614G, Delta and Omicron variants.^13^ Lower anti-spike titres, and thus a lower neutralising capacity against SARS-CoV-2, could potentially lead to more breakthrough infections.^28^ Despite our study not being powered to estimate relative vaccine effectiveness, we observed no differences in SARS-CoV-2 infections by vaccine strategy in our cohort. Further randomised control trials are needed for confirmation of non-inferior immunogenicity following co-administration. The 20% reduction in post-booster anti-spike titres seen in our study, if observed in a randomised trial, would not meet the preferred non-inferiority margin of 0.67 or 0.5 which is often used for vaccine licensing.^31^^,^^32^ If there is definitive evidence of a reduction in vaccine effectiveness with concomitant vaccination, the impact of the potential reduced vaccine coverage that may result from changes to the vaccine schedule would need to be evaluated. Co-administration of vaccines has been an effective way of increasing uptake of both SARS-CoV-2 booster and influenza vaccinations.^33^ Changing to separate administration, due to a small reduction in immunogenicity, is unlikely to be beneficial if it results in reduced vaccine uptake, and would need to be implemented with care given the barriers to the delivery of vaccines, and the hesitancy for regular SARS-CoV-2 vaccination amongst subgroups of HCWs.^34^

To the best of our knowledge this is the first study to show there is no difference in T cell response to SARS-CoV-2 by vaccine strategy and this was observed throughout the study period. Recent studies have shown that the adaptive cellular immune response may play a role in preventing initial infection.^35^^,^^36^ There are no definitive antibody or cellular immune responses that accurately correlates with protection against SARS-CoV-2 and the modest reduction in peak antibody titre following concomitant vaccination may not have an impact on vaccine effectiveness if the cellular immune response can overcome this deficit. Of note, there were no differences in influenza HAI titres by vaccination strategy, which is consistent with previous studies.^14^ Further research is required to examine if the cellular response to IIV are impacted by concomitant vaccination.

Our study has limitations. Firstly, unlike a majority of studies that have evaluated the impact of concomitant vaccination, we conducted an observational study which meant that there may be unmeasured differences between the two groups beyond age and sex. There were differences in baseline characteristics of those included and excluded from the analysis. This is likely due to the exclusion of participants that did not receive the influenza vaccine, healthcare workers not receiving the vaccine tend to be younger, from ethnic minority groups and less likely to have received a previous influenza vaccine. In addition, there was greater non-attendance at the 1-month post-vaccine visit amongst those vaccinated separately since this required an additional study visit and blood test.

Secondly, our study follow-up period coincided with a rapid increase in SARS-CoV-2 transmission due to the emergence of the Omicron variant,^37^ which resulted in 50% of participants becoming infected and therefore being excluded from our 6-months post-booster analysis. By excluding those that became infected, we are unable to examine whether these individuals would have exhibited different immune responses at 6-months post-vaccination had they not become infected. In addition, due to the large number of exclusions our study may have been underpowered to find differences in immune responses at 6-months post-vaccination.

Lastly, our study was not designed to examine the post-vaccination anti-spike antibody trajectory, since we only sampled at two post-vaccination time points. We were therefore unable to evaluate anti-spike antibody persistence or the exact timing of peak antibody responses. It is possible that antibody responses peaked to the same titre in the two vaccination groups, but that these peaks occurred after different periods of time post-vaccination. Further studies with more frequent sampling in the first month and during the 6-months post-vaccination are required to better characterise post-vaccination antibody kinetics and delineate the impact vaccination strategy has on these trajectories.

In conclusion, our study adds to the growing body of evidence that suggests there is a difference in the peak humoral response to the SARS-CoV-2 booster and preserved peak influenza HAI titres when given concomitantly with the IIV. Further work is needed to establish whether small differences in post-vaccination anti-SARS-CoV-2 antibody titres translate to different clinical outcomes. Encouragingly, this study is the first to show that these differences do not persist, and there were no significant differences in SARS-CoV-2 cellular immune response by vaccination strategy. Our study has urgent public health implications, in observing that concomitant vaccination with SARS-CoV-2 and influenza vaccines has no significant impacts on long-term immunogenicity. Further studies are needed to evaluate anti-spike trajectories, the impact this would have on vaccine effectiveness and determine if this would outweigh the reduced vaccine coverage that may result from changes to the vaccine schedule.

## Contributors

MP conceived the ideas for BE-DIRECT and led the applications for funding with input from CAM, JN, PH and AC. Online consent and questionnaire tools were designed and implemented by CAM and LB. Recruitment for the studies was done by CAM, JN, DP, NV, DV, VR, AK, PR and MP. Sample and additional data collection was done by CAM, JN, VR and DV. Influenza HAI assays were performed by HP. SARS-CoV-2 anti-spike antibody assays were performed by ADO. ELISpot assays were performed by AT laboratory. Data were linked and cleaned by CAM with input from JN, DP, VR, DV, and MP. Data analysis was completed by JN and CAM with input from DP, MD, JWT, LJG, LT, PH, AC, SGS and MP. JN wrote the first draft of the manuscript with input from CAM, DP, JWT, IGB, SGS, PM, IS, MJW, and NV, all other authors reviewed the manuscript and provided comments. All authors have had the opportunity to access the underlying data used in this study. All authors reviewed the manuscript and approved the final version prior to submission.

## Data sharing statement

To access data or samples produced by the BE-DIRECT, the working group representative must first submit a request to the Core Management Group by contacting the UK-REACH Project Manager in the first instance. For ancillary studies outside of the core deliverables, the Steering Committee will make final decisions once they have been approved by the Core Management Group. Decisions on granting the access to data/materials will be made within eight weeks. Third party requests from outside the Project will require explicit approval of the Steering Committee once approved by the Core Management Group.

## Declaration of interests

PM has received honoraria from Moderna, BioNTech, Gilead, AstraZeneca and GSK, support for attending meetings from AstraZeneca and has participated on an advisory board for AstraZeneca and Moderna. IB declares shares in an influenza vaccine manufacturing company. PH received an honorarium for hosting a COVID-19 webinar, on behalf of Oxford Immunotec (now Revvity) who are manufacturers of the ELISpot technology used in the manuscript. AT is an employee of Revvity. MP reports grants from UKRI-MRC for the current work and UKRI-MRC, NIHR, Sanofi, Gilead and Moderna outside the current work and has received consulting fees from QIAGEN. SGS reports consultancy or advisory role for CSL Seqirus, Novavax, Moderna, Sanofi and Evo Health. The WHO Collaborating Centre for Reference and Research on Influenza received funding from the International Federation of Pharmaceutical Manufacturers and Associations and from CSL Seqirus for the production of influenza vaccines. DP is supported by a NIHR Doctoral Research Fellowship. The views expressed are those of the author(s) and not necessarily those of the NIHR or the Department of Health and Social Care.
