# Supplementary material for: Immunogenicity of concomitant SARS-CoV-2 and influenza vaccination in UK healthcare workers: a prospective longitudinal observational study
Source: Lancet Reg Health Eur. 2024 Aug 12;44:101022. doi: 10.1016/j.lanepe.2024.101022 (PMC11496956; doi:10.1016/j.lanepe.2024.101022)
Supplement: Supplementary Text, Tables and Figures [file mmc1.docx]

**Immunogenicity of concomitant SARS-CoV-2 and Influenza vaccination in UK healthcare workers: a prospective longitudinal observational study**

Joshua Nazareth^1,2,3,4,*^, Christopher A. Martin^1,2,3,4,*^, Daniel Pan^1,2,3,4,5^, Ian G. Barr^6^, Sheena G. Sullivan^6,7,8^, Heidi Peck^6^, Neyme Veli^1,^, Mrinal Das^1^, Luke Bryant ^1,3^, Nisha George^1,2,3,4^, Marjan Gohar^1,2,3,4^, Laura J. Gray^9^, Lucy Teece^9^, Denny Vail^10^, Val Renals^10^, Aleesha Karia^10^, Paul Renals^10^ Paul Moss^11^, Andrea Tattersall^12^, Ashley D. Otter^13^, Pranab Haldar^1,14^, Andrea Cooper ^1,3^, Iain Stephenson^2^, Martin J. Wiselka^1,2^, Julian W. Tang^1,15^, Laura Nellums^16,17^, Manish Pareek^1,2,3,4,^^

^1^Department of Respiratory Sciences, University of Leicester, UK

^2^Department of Infection and HIV Medicine, University Hospitals of Leicester NHS Trust, Leicester, UK

^3^Leicester NIHR Biomedical Research Centre, Leicester, UK

^4^Development Centre for Population Health, University of Leicester, Leicester, UK

^5^Li Ka Shing Centre for Health Information and Discovery, Oxford Big Data Institute, University of Oxford, UK

^6^WHO Collaborating Centre for Reference and Research on Influenza, Royal Melbourne Hospital, at The Peter Doherty Institute for Infection and Immunity, Melbourne, Australia

^7^Department of Infectious Diseases, University of Melbourne, Melbourne, Australia

^8^Department of Epidemiology, University of California, Los Angeles, USA

^9^Department of Population Health Sciences, University of Leicester, UK

^10^Research Space, University Hospitals of Leicester NHS Trust, UK

^11^Institute of Immunology and Immunotherapy, University of Birmingham, Birmingham, UK

^12^Revvity (Certimmune), Abingdon, Oxfordshire, UK

^13^UK Health Security Agency, Porton Down, Salisbury, UK

^14^Department of Respiratory Medicine, University Hospitals of Leicester NHS Trust, Leicester, UK

^15^Department of Clinical Microbiology, University Hospitals of Leicester NHS Trust, Leicester, UK

^16^Lifespan and Population Health Academic Unit, School of Medicine, University of Nottingham, Nottingham, UK

^17^College of Population Health, University of New Mexico, New Mexico, USA

* Joint first authors

**^ Corresponding authors:**

Prof. Manish Pareek

Department of Respiratory Sciences,

University of Leicester,

Leicester, UK

Email: [mp426@le.ac.uk](mailto:mp426@le.ac.uk)

**Table of contents**

Supplementary text………………………………………………………………………… 3

Supplementary figures..….………………………………………………………………… 5

Supplementary tables.……………………………………………………………………… 6

References...………...……………………………………………………………………… 15

**Supplementary Text 1. Study design**

This study was conducted at University Hospitals of Leicester NHS Trust (UHL) as part of the BE-DIRECT (Broadening our understanding of Early versus Late InfluEnza Vaccine Effectiveness - Determining the Immune Response in Ethnic minority healthcare workers to COVID-19 infecTion) study, an ongoing single-centre prospective cohort study evaluating the differences in immune responses to SARS-CoV-2 infection and vaccination by ethnicity (see Supplementary Text 1 for further details). We conducted this component of the prospective cohort study between 29th September 2021 and 5th August 2022. We recruited HCWs aged 16 years or over who worked at UHL and in the surrounding area. HCWs could participate regardless of previous influenza and SARS-CoV-2 infections or vaccinations. For the purpose of this study, we used the term HCW to include any staff member with or without direct patient contact, as well as healthcare students and volunteers.

**Supplementary Text 2. Methodology for SARS-CoV-2 serology assay**

Anti-spike and anti-nucleocapsid SARS-CoV-2 serology were performed at UKHSA Porton Down on serum samples using the Roche Elecsys anti-SARS-CoV-2 S (Product code: 09203079190) and Roche Elecsys anti-SARS-CoV-2 (Product code: 09289275190) assays,^1^ which utilise the Wuhan receptor binding domain and so were homologous to the mRNA SARS-CoV-2 vaccine (BNT162b2 /Comirnaty, Pfizer-BioNTech). Assays showed high reproducibility, high sensitivity and high specificity. Samples were considered positive for anti-spike antibodies if ≥ 0.8 binding antibody units per millilitre (BAU/ml), and positive for anti-nucleocapsid antibodies if ≥ 1 cutoff index (COI). All samples were tested within 1-2 days of one another, using the same lot number and staff member. As discussed in the UKHSA evaluation of the Roche assay,^1^ inter and intra-variability of the assay is extremely low, producing highly reproducible data.

**Supplementary Text 3. Methodology for Influenza haemagglutination inhibition (HAI) assay**

The HAI assays were performed at the World Health Organization (WHO) Collaborating Centre for Reference and Research on Influenza, Melbourne, Australia according to the WHO method.^2^ Viruses used for the HAI assay were identical to those used in the vaccine. All viruses were cell propagated and diluted to 4 HA units/25 µl prior to use and the B-viruses were ether split before use.^3^ Sera were pre‐treated with receptor destroying enzyme (Denka‐Seiken, Japan) according to the manufacturer’s instructions before enzyme inactivation by addition of an equal volume of 1.6% trisodium citrate and incubation at 56°C for 30 minutes. Treated sera were diluted in a 2-fold series starting at 1:10 and 25 µl of virus was added for 45 minutes and incubated at room temperature, followed by the addition of 25 µl of 1% (v/v) turkey red blood cells (or guinea pig red blood cells for H3 viruses) and incubated for a further 30 minutes. All subjects pre- and post-vaccination sera were tested on the same run, and on the same HI plate. We tested reproducibility by testing a set panel of sera and viruses using different operators. We also tested accuracy by repeating the same assay on different days. The serum titre was expressed as the reciprocal of the highest serum dilution that caused complete inhibition of hemagglutination.

**Supplementary Text 4. Methodology for defining SARS-CoV-2 infection during the study period**

We defined evidence of SARS-CoV-2 infection in the pre-vaccination to 1-month post-vaccination period as a fourfold increase in anti-nucleocapsid titre between timepoints. Additionally, having a twofold increase in anti-nucleocapsid titre with evidence of a higher ELISpot count in response to SARS-CoV-2 nucleocapsid or membrane peptides at the 1-month post-vaccine visit compared to the pre-vaccine visit was considered as evidence of infection.

In the 1-month post-vaccination to 6-months post-vaccination period, we defined evidence of SARS-CoV-2 infection as anti-nucleocapsid seroconversion between the 1-month and 6-months post-booster timepoints. For those who were already seropositive for anti-nucleocapsid antibodies, we considered a higher anti-nucleocapsid antibody titre at 6-months compared to 1-month post-booster, or a higher ELISpot count in response to nucleocapsid or membrane peptides at 6-months compared to 1-month post-booster, with a concurrent rise in anti-spike antibody titre as evidence of infection.

**Supplementary Text 5. Methodology for defining ELISpot counts**

Raw ELISpot counts were transformed using the same methodology in our previous work.^4^ A nil control was used to measure background T cell activation and the spot count from the unstimulated sample was subtracted from the values after stimulation to account for it. The nil control was completed using the addition of only culture media to the sample.

**Supplementary Figure 1.** **Schedule of study visits.** Figure illustrating the schedule of study visits and timing of sample collection. If participants were vaccinated concomitantly samples from visits 2 and 3 were collected at the same time. The order of visits 2 and 3 was dependent on the order that participants received the IIV and SARS-CoV-2 booster. IIV, inactivated influenza vaccine.

**Supplementary Table 1. Baseline characteristics for each analysis.** Baseline characteristics of the participants included in each analysis by vaccination strategy, influenza vaccine and SARS-CoV-2 booster administered concomitantly or separately. All results are shown as n (%) unless otherwise stated with percentage being presented column-wise. IQR, Interquartile range; VC Vaccinated concomitantly, VS Vaccinated separately.

**Supplementary Table 2.** Baseline characteristics of the participants included and excluded in each analysis. All results are shown as n (%) unless otherwise stated with percentage being presented column-wise. IQR, Interquartile range; n/a, not applicable; * Significant difference for that demographic or clinical feature between included and excluded *p* value <0.05

**Supplementary Table 3.** **Linear regression models showing the association between vaccine strategy and other parameters with ln SARS-CoV-2 post-booster anti-spike antibody titre.** The table shows the exponentiated coefficients to give an adjusted geometric mean ratio (aGMR) with corresponding 95% confidence intervals for the multivariable linear regression simplified model (model 1) and fully adjusted model (model 2) with an outcome of ln SARS-CoV-2 anti-spike antibody titre (BAU/ml) 1-month post-booster (top) and 6-months post-booster (bottom), as seen in Figure 3. GMR, geometric mean ratio.

**Supplementary Table 4.** **Linear regression models showing the association of time between IIV and SAR-CoV-2 booster and other parameters with ln SARS-CoV-2 post-booster anti-spike antibody titre.** The table shows the exponentiated coefficients to give an adjusted geometric mean ratio (aGMR) with corresponding 95% confidence intervals for the multivariable linear regression models that included only those that were vaccinated separately. The simplified model (model 1) just adjusted for number of weeks between vaccination and pre-booster anti-spike titre and model 2 is a fully adjusted model, both have an outcome of natural logarithm (ln) SARS-CoV-2 anti-spike antibody titre (BAU/ml) post-booster.

**Supplementary Table 5. Linear regression models restricted to participants that did not become infected by 6-months post-booster, showing the association between vaccine strategy and other parameters with ln SARS-CoV-2 post-booster anti-spike antibody titre.** The table shows the exponentiated coefficients to give an adjusted geometric mean ratio (aGMR) with corresponding 95% confidence intervals for the multivariable linear regression simplified model (model 1) and fully adjusted model (model 2) with an outcome of ln SARS-CoV-2 anti-spike antibody titre (BAU/ml) 1-month post-booster. GMR, geometric mean ratio.

**Supplementary Table 6. Negative binomial regression model showing the association between vaccine strategy and other parameters with S1, S2 and Spike specific T cell responses 1-month post-booster.** The table shows the adjusted incidence rate ratios (aIRRs) with corresponding 95% confidence intervals for the negative binomial regression simplified model (model 1) and fully adjusted model (model 2) with an outcome of ELISpot count in response to peptides derived from SARS-CoV-2 S1, S2 and spike (S1 + S2) 1-month post-booster, as seen in Figure 4. IRR, incidence rate ratio.

**Supplementary Table 7.** **Negative binomial regression model showing the association between vaccine strategy and other parameters with S1, S2 and Spike specific T cell responses 6-months post-booster.** The table shows the adjusted incidence rate ratios (aIRRs) with corresponding 95% confidence intervals for the negative binomial regression simplified model (model 1) and fully adjusted model (model 2) with an outcome of ELISpot count in response to peptides derived from SARS-CoV-2 S1, S2 and spike (S1 + S2) 6-months post-booster, as seen in Figure 4. IRR, incidence rate ratio.

**Supplementary Table 8.** **Linear regression models showing the association between vaccine strategy and other parameters with ln HAI 1-month post-vaccine titre.** The table shows the exponentiated coefficients to give an adjusted geometric mean ratio (aGMR) with corresponding 95% confidence intervals for the multivariable linear regression simplified model (model 1) and fully adjusted model (model 2) with an outcome of natural logarithm (ln) HAI titre 1-month post-vaccine for each influenza strain, as seen in Figure 6. GMR, geometric mean ratio; HAI, haemagglutinin inhibition assay.

**Supplementary Table 9.** **Linear regression models showing the association between vaccine strategy and other parameters with ln HAI 6-months post-vaccine titre.** The table shows the exponentiated coefficients to give an adjusted geometric mean ratio (aGMR) with corresponding 95% confidence intervals for the multivariable linear regression simplified model (model 1) and fully adjusted model (model 2) with an outcome of ln HAI titre 6-months post-vaccine for each influenza strain, as seen in Figure 6. GMR, geometric mean ratio; HAI, haemagglutinin inhibition assay.

**References**

1. England PH. COVID-19: laboratory evaluations of serological assays 2021 [Available from: <https://www.gov.uk/government/publications/covid-19-laboratory-evaluations-of-serological-assays>.

2. World Health O. Manual for the laboratory diagnosis and virological surveillance of influenza. Geneva: World Health Organization; 2011.

3. Jennings R, Clark A, Oxford JS, Hockley DJ, Potter CW. Reactogenicity and immunogenicity of whole and ether-Tween-split influenza A virus vaccines in volunteers. J Infect Dis. 1978;138(5):577-86.

4. Martin CA, Nazareth J, Jarkhi A, Pan D, Das M, Logan N, et al. Ethnic differences in cellular and humoral immune responses to SARS-CoV-2 vaccination in UK healthcare workers: a cross-sectional analysis. eClinicalMedicine. 2023;58.
